# Endoscopic ultrasound-guided antegrade dilation using a drill dilator for a pancreatojejunostomy anastomotic stricture, with pancreatoscopic findings

**DOI:** 10.1055/a-2055-1306

**Published:** 2023-04-11

**Authors:** Takeshi Ogura, Junichi Nakamura, Jun Sakamoto, Yuki Uba, Hiroki Nishikawa

**Affiliations:** 1 Endoscopy Center, Osaka Medical and Pharmaceutical University Hospital, Osaka, Japan; 2 2nd Department of Internal Medicine, Osaka Medical and Pharmaceutical University, Osaka, Japan

Pancreaticojejunal anastomotic stricture (PJS) occurs as a complication after pancreaticoduodenostomy, with a reported incidence rate of 1.4 %–11.4 % 1 . Because abdominal pain or acute pancreatitis can occur in patients with a PJS, resolution of the PJS is desirable. The endoscopic approach is more often selected because, compared with surgical treatment, it is a noninvasive procedure. Although the enteroscopic approach is usually attempted 2 3 , it has disadvantages such as prolonged procedure time. Accordingly, endoscopic ultrasound-guided pancreatic duct drainage (EUS-PD) has been developed 4 . This alternative method is reported to have a high technical success rate and requires dilation of the PJS. The Tornus ES drill dilator (Asahi Intecc) ( Fig. 1 ) has recently become available in Japan 5 . It enables the tract to be easily dilated using a clockwise rotation without any pushing force. We herein describe its successful use for dilation of a PJS, with the pancreatoscopic findings shown.

**Fig. 1:**
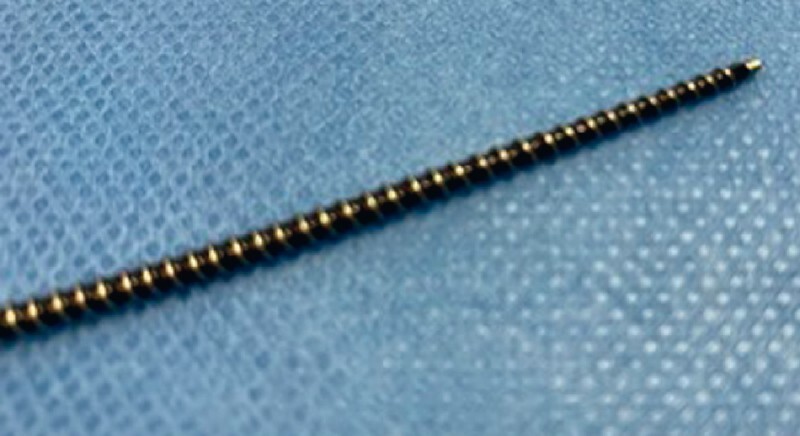
Photograph of the Tornus ES drill dilator (Asahi Intecc).

A 77-year-old man was admitted to our hospital with frequent episodes of acute pancreatitis. He had undergone pancreaticoduodenostomy for pancreatic head cancer 6 months previously. Computed tomography revealed a PJS, and EUS-PD with a plastic stent was performed. After 3 months, treatment was attempted for the PJS. The fistula was dilated using a balloon catheter ( Fig. 2 ), following which the PJS site was evaluated after antegrade insertion of a pancreatoscope. Pancreatoscopy revealed a tight stricture, but no findings to indicate recurrence of the pancreatic cancer ( Fig. 3 ). Because, after a guidewire had been deployed, the endoscopic retrograde cholangiopancreatography catheter could not be inserted into the intestine across the PJS, PJS dilation was attempted using the drill dilator and was easily achieved ( Fig. 4 ). Further pancreatoscopy showed resolution of the PJS without any bleeding ( Fig. 5 ; Video 1 ). No recurrence of the PJS has been observed during the 12 months following this procedure.

**Fig. 2:**
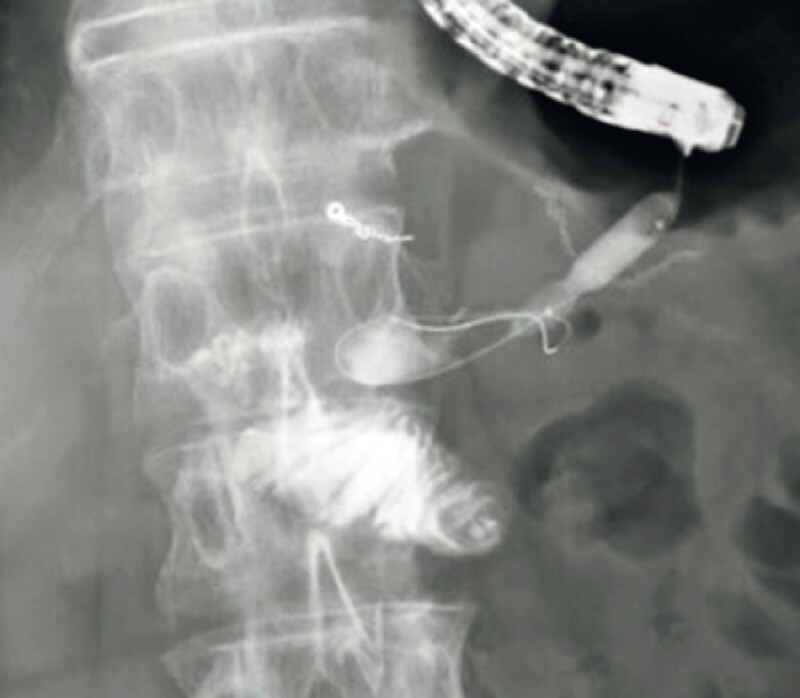
Fluoroscopic image showing the fistula being dilated using a balloon catheter.

**Fig. 3:**
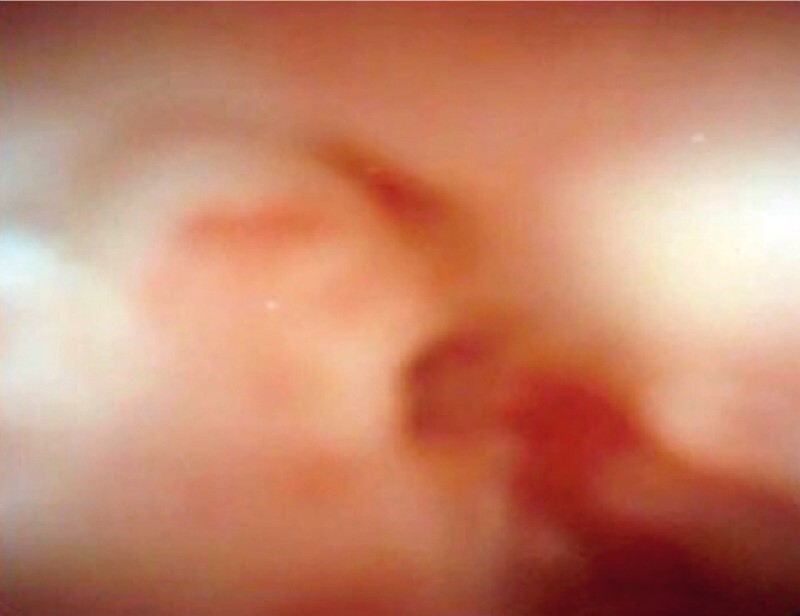
Pancreatoscopic view of the site of the pancreaticojejunal anastomotic stricture showing a tight stricture, but no findings to indicate recurrence of the pancreatic cancer.

**Fig. 4:**
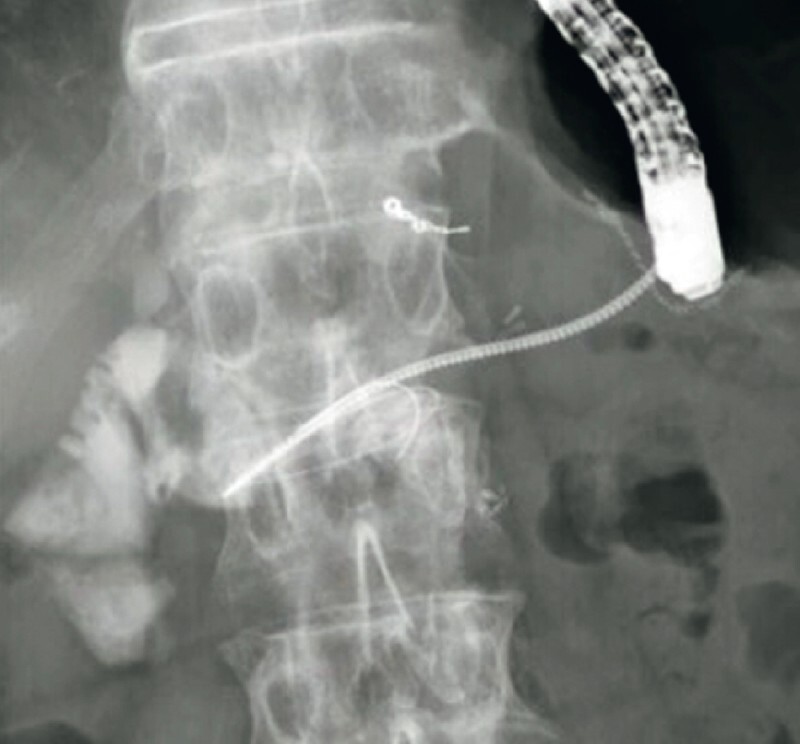
Fluoroscopic image showing dilation of the pancreaticojejunal anastomotic stricture being attempted and easily achieved using the drill dilator.

**Fig. 5:**
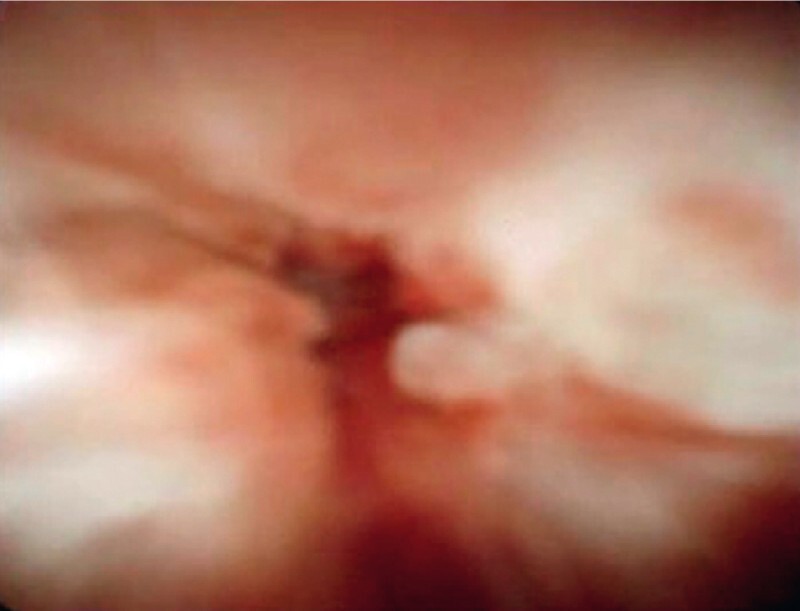
Final pancreatoscopic image showing resolution of the pancreaticojejunal anastomotic stricture with no evidence of bleeding.

**Video 1** Endoscopic ultrasound-guided antegrade dilation using a drill dilator for a pancreatojejunostomy anastomotic stricture, with pancreatoscopic findings shown.


In conclusion, the Tornus ES drill dilator may be useful in obtaining resolution of a PJS; however, additional studies with more cases are required.

Endoscopy_UCTN_Code_TTT_1AS_2AD

## Notes

Endoscopy E-Videos

https://eref.thieme.de/e-videos
*E-Videos* is an online section of the journal *Endoscopy* , reporting on interesting cases and new techniques in gastroenterological endoscopy. All papers include a high-quality video and are published with a Creative Commons CC-BY license. Endoscopy E-Videos qualify for HINARI discounts and waivers and eligibility is automatically checked during the submission process. We grant 100% waivers to articles whose corresponding authors are based in Group A countries and 50% waivers to those who are based in Group B countries as classified by Research4Life (see: https://www.research4life.org/access/eligibility/ ). This section has its own submission website at https://mc.manuscriptcentral.com/e-videos
